# Are Pregnant Women Who Are Living with Overweight or Obesity at Greater Risk of Developing Iron Deficiency/Anaemia?

**DOI:** 10.3390/nu13051572

**Published:** 2021-05-07

**Authors:** Anna A. Wawer, Nicolette A. Hodyl, Susan Fairweather-Tait, Bernd Froessler

**Affiliations:** 1 Department of Anaesthesia, Lyell McEwin Hospital, Elizabeth Vale, SA 5112, Australia; anna.wawer@sa.gov.au; 2 Faculty of Health and Medical Sciences, Adelaide Medical School, University of Adelaide, Adelaide, SA 5005, Australia; nicolette.hodyl@adelaide.edu.au; 3 Norwich Medical School, University of East Anglia, Norwich Research Park, Norwich NR4 7TJ, UK; s.fairweather-tait@uea.ac.uk; 4 Discipline of Acute Care Medicine, Adelaide Medical School, University of Adelaide, Adelaide, SA 5005, Australia

**Keywords:** pregnancy, anaemia, iron deficiency, overweight, obesity, inflammation

## Abstract

Low-grade inflammation is often present in people living with obesity. Inflammation can impact iron uptake and metabolism through elevation of hepcidin levels. Obesity is a major public health issue globally, with pregnant women often affected by the condition. Maternal obesity is associated with increased pregnancy risks including iron deficiency (ID) and iron-deficiency anaemia (IDA)—conditions already highly prevalent in pregnant women and their newborns. This comprehensive review assesses whether the inflammatory state induced by obesity could contribute to an increased incidence of ID/IDA in pregnant women and their children. We discuss the challenges in accurate measurement of iron status in the presence of inflammation, and available iron repletion strategies and their effectiveness in pregnant women living with obesity. We suggest that pre-pregnancy obesity and overweight/obese pregnancies carry a greater risk of ID/IDA for the mother during pregnancy and postpartum period, as well as for the baby. We propose iron status and weight gain during pregnancy should be monitored more closely in women who are living with overweight or obesity.

## 1. Introduction 

This review explores inflammation as a potential underlying cause of iron deficiency (ID) or iron-deficiency anaemia (IDA) in pregnant women who are living with overweight or obesity. The aim of this review is to summarise results from studies conducted worldwide in the last ten years that report measurements of iron and inflammatory status in pregnant women who are living with overweight or obesity. Finally, this review will evaluate current treatments for ID/IDA in this same context.

For the investigations on the effect of inflammation in pregnant women who are living with overweight or obesity on iron status, the Pubmed library was used. The following search terms were used:

1. Overweight, obes*, BMI;
2. Mother*, maternal, preg*, neonat*;
3. Anaem*, anem*iron status, ferritin, hepcidin;
4. Inflamm*.

For example, overweight AND mother* AND anaem* AND inflamm*.

The search yielded 339 studies; 305 duplicates and 20 studies that did not focus on pregnancy were removed, leaving 14 that are discussed below in greater detail.

## 2. Iron Deficiency and Iron-Deficiency Anaemia in Pregnancy

Anaemia is a condition characterised by a low haemoglobin concentration. Iron deficiency is responsible for approximately 50% and 60% of anaemia and severe anaemia, respectively, in pregnant women [1]. According to the World Health Organisation (WHO), anaemia is defined as haemoglobin levels <110 g/L for pregnant women [2]. Haemoglobin thresholds that define severe anaemia are <70 g/L for pregnant women and <80 g/L for non-pregnant women. Worldwide, 38% of pregnant women suffer from anaemia [3]. In Australia, the estimates of anaemia prevalence are 25% for pregnant women and 15% for children aged 6–59 months [2].

Iron requirements and iron absorption change across pregnancy, with a fall observed in the first trimester. However, by the third trimester, iron requirements and absorption increase more than three-fold [4] due to increased placental demand, fetal growth, maternal red blood cell mass expansion, and repletion of maternal iron stores [5]. Changes in iron biology in pregnancy are presented by trimester in Table 1 [5,6].

**Figure 1:**
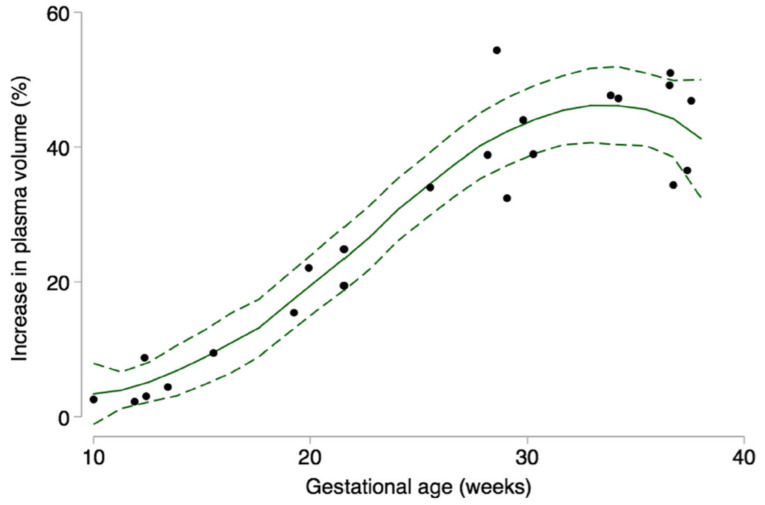
Percent plasma volume expansion across gestation compared to non-pregnant state, reported in a systematic review by Aguree and Gernard [8], used with permission of the original publisher, Springer Nature Group, “Dots represent data from individual studies; solid line represents prediction based on all data; short dashed line represents the 95% CI around the prediction”.

Plasma volume expansion (Figure 1) starts towards the end of the first trimester, followed by both plasma volume and red blood cell mass expansion in the second and third trimester. Overall plasma volume increase throughout the pregnancy is greater than the red blood cell mass growth, with the total increase being approximately 50% (plasma) and 35% (red blood cell mass) [6].

A compelling report by Ferguson et al. [9] has questioned the use of current WHO cutoffs for anaemia in pregnancy. The authors state that the reference ranges for haemoglobin in pregnancy are based on two small studies (non-blinded, non-randomized) published 5 decades ago. Given that approximately 45% of women of childbearing age are iron deficient [10], these studies inevitably included women with low or absent iron stores, thus potentially driving the haemoglobin reference ranges down. The authors also point out that the effect of haemodilution (i.e., red blood cell mass growth not keeping up with plasma volume expansion) may be due to insufficient iron supply. This is supported by studies showing a reduction in haemodilution when iron supplements are provided [11,12].

IDA during pregnancy can result in preterm labour, low birth weight, and increased risk of anaemia in infants after 4 months of age. Anaemic mothers also experience tiredness, headaches, lethargy and decreased work and exercise tolerance, all of which negatively affect the quality of life and well-being. Severe IDA can result in increased risk of maternal and infant mortality [13,14,15].

At birth, a significant proportion of iron is in the form of fetal haemoglobin, which has higher iron content; this is all converted to adult haemoglobin by the age of 10–12 weeks, and the surplus iron recycled for re-use. Very rapid growth during infancy quickly drains iron reserves accumulated by the fetus during gestation, frequently resulting in child iron deficiency [16]. Iron stores are depleted by approximately 6 months of age and if there is an inadequate supply of iron, ID/IDA [17] develops. This can result in compromised behavioural, cognitive and motor development, with irreversible consequences [18,19,20]. The prevalence of ID/IDA in pregnancy and its effect on iron status and neurocognitive deficits of the infant have been discussed in detail elsewhere [6,21].

Rates of iron deficiency and IDA in children are high, particularly in the Indigenous Australian population. For example, 68% Indigenous Australian infants from northern Australia aged between 6 and 12 months were found to be anaemic [22]. This is much higher than the non-Indigenous Australian population, where 10% of infants and children below 2 years of age were iron deficient and 3% were anaemic [23]. Atkins et al. [24] have also reported that in Melbourne, approximately 19% of toddlers and 33% of infants had inadequate iron intakes.

Despite major scientific efforts worldwide in its prevention and/or correction, ID/IDA still remains a significant global challenge [3] and is in the top five causes for years lived with disability [3].

## 3. Does Obesity in Pregnancy Trigger an Inflammatory Response?

### 3.1. Prevalence of Obesity in Pregnancy

Obesity is another major public health issue worldwide that is increasing in prevalence [25,26]. Obesity is defined as a body mass index (BMI) >30 kg/m^2^ and classified as a disease by the WHO. Obesity is a risk factor for a range of diseases, including high blood pressure, stroke, heart disease, type 2 diabetes, certain types of cancer and arthritis [27,28]. Further, obesity decreases quality of life, strains health care systems and society [27]. In pregnancy, a BMI of 30 kg/m^2^ and above in the first trimester is defined as obesity, a BMI of 25.0–29.9 kg/m^2^ as overweight, and a BMI of 18.5–24.9 kg/m^2^ as normal [29]. Obesity categories are divided into class I (BMI of 30 and <35), class II (BMI of 35 and <40) and class III (BMI of 40 and higher). A study from 2010 (from South Australia) reported that 35.9% and 16.7% of women who gave birth were overweight and living with obesity, respectively [30]. Currently in Australia, almost 50% of women who give birth are either overweight or obese [31,32].

Recommended weight gain in pregnancy as well as rates of weight gain vary between different pre-pregnancy BMI groups and are presented in Table 2 below.

However, despite clear weight gain goals, almost half of pregnant women gain more weight than is recommended, with women in the overweight and obese BMI categories most frequently exceeding the optimal ranges [34].

Maternal obesity is associated with an increased risk of gestational diabetes [35,36], pre-eclampsia, fetal intrauterine growth restriction, congenital abnormalities, fetal complications, stillbirth, infant death [37,38], preterm birth, caesarean delivery and greater risk of low Apgar score [39,40]. Maternal obesity also increases the likelihood of childhood obesity and type 2 diabetes in the future in mothers and their children [35,41,42]. In her review, Leddy et al. [41] describes in detail obstetric complications associated with obesity in pregnancy.

A further increase in the prevalence of obesity in adults is expected, as overweight and obese children are more likely to become obese adults [43]. Not surprisingly, 26.3% of young Australian girls were in the overweight/obese category [43], thus the number of women of childbearing age who are living with overweight or obesity will also increase in the future.

### 3.2. Inflammatory Status in Healthy Pregnancies

Physiologically, an altered inflammatory state during pregnancy is crucial for successful implantation of the embryo and for positive pregnancy outcome [44,45,46,47]. There are several reports in the literature describing the dynamics of inflammation during pregnancy but they exhibit a large degree of inconsistency. Some researchers report a progressive increase in CRP throughout pregnancy [48,49,50]. Others indicate that the first and third trimesters (together with delivery phase) [44,51] are pro-inflammatory, while the second trimester is an anti-inflammatory [44] phase. Belo et al. [47] reported large variations in CRP levels between/within pregnancy trimesters in his subjects, whereas Christian et al. [52] reported a progressive decrease in CRP during pregnancy. Finally, racial differences in CRP values during pregnancy have also been reported [53].

### 3.3. Obesity, the Inflammatory Response and Iron Status

Pre-pregnancy obesity has been identified as an independent risk factor for postpartum anaemia [54,55]. As reported by Bodnar et al. the relative risk of postpartum anaemia increased with an increase in pre-pregnancy BMI (the relative risk increased 1.8-fold for BMI ≥ 28 and 2.8-fold for BMI ≥ 36 when compared with individuals with BMI = 20) [56].

Hepcidin is a key regulator of iron metabolism in the body, and has been identified as one of the links between obesity and ID/IDA [57,58]. Specifically, both increased inflammation and expression of leptin stimulate hepcidin production in the liver [59,60]. Elevated hepcidin then binds to iron loaded ferroportin (present in enterocytes or in the plasma membrane of macrophages) causing its internalisation and degradation. Consequently, iron efflux into the circulation is prevented [61], a state which leads to anaemia of inflammation (AI). AI can occur despite sufficient body iron stores (ferritin) due to the direct actions of hepcidin on ferroportin [62]. Erythroferrone is a recently discovered hormone that acts as a suppressor of hepcidin and directly links erythropoiesis and iron metabolism [63,64]. During increased erythropoiesis (in response to hemorrhage, for example), erythroblasts release erythroferrone which reduces hepcidin expression, thereby permitting the release of cellular iron from stores as well as increasing iron uptake from the gut [63,64]. Whether erythroferrone action on hepcidin remains unchanged in inflammatory states or blood volume expansion during pregnancy remains to be established

Interleukin 6 (IL-6) is a pro-inflammatory cytokine, responsible for the induction of a generalised inflammatory response which then triggers hepcidin production in the liver [59,60]. While IL-6 can be produced by activated immune cells, it is also produced by adipose tissue. Studies have demonstrated that adipose tissue produces approximately 30% of circulating IL-6 [65,66]. Among other more direct actions, elevated IL-6 causes the liver to produce CRP [67].

Ferritin is an iron storage protein as well as an acute-phase protein. The blood concentration rises rapidly in response to inflammation, when it is no longer representative of iron stores. In the first 24 h of onset of inflammation, ferritin and CRP rise in a similar fashion; with CRP levels peaking at approximately 48 h [68,69]. Ferritin levels peak at approximately 96 h from the onset of inflammation and remain elevated in a similar way to another inflammatory protein, alpha-1 acid glycoprotein (AGP), whereas CRP levels decrease more rapidly. Six days (final timepoint of the observation) after the onset of inflammation, all three proteins—ferritin, AGP and CRP—continue to remain elevated well above the pre-inflammatory concentrations (150%, 75% and 25% above the baseline for ferritin, AGP and CRP, respectively) [68,69,70].

One of the pathways through which obesity may negatively affect iron status in pregnancy is through low-grade inflammation [71,72], a chronic state that may lead to AI. Specifically, this may result from elevated inflammatory proteins and cytokines, including CRP, IL-6, TNF-alpha and AGP [73]. This has been observed in non-pregnant individuals who are living with overweight or obesity [52]. Alternatively, poor iron status may occur as a result of a diet low in bioavailable iron and thus an inability to meet the increased iron demands of pregnancy.

Enlarged adipocytes release higher levels of cytokines and leptin [57,74] and lower levels of the anti-inflammatory hormone adiponectin Thus, increased adipose tissue mass directly contributes to an increase in low-level chronic inflammation [65,73,75]. A detailed summary and description of inflammatory mediators in maternal obesity was reported in 2017 in a review by Pendeloski et al. [76]. Despite the large number of studies available, contradictory results on the profile of inflammatory mediators in pregnant women who are living with overweight or obesity have been reported.

**Figure 2:**
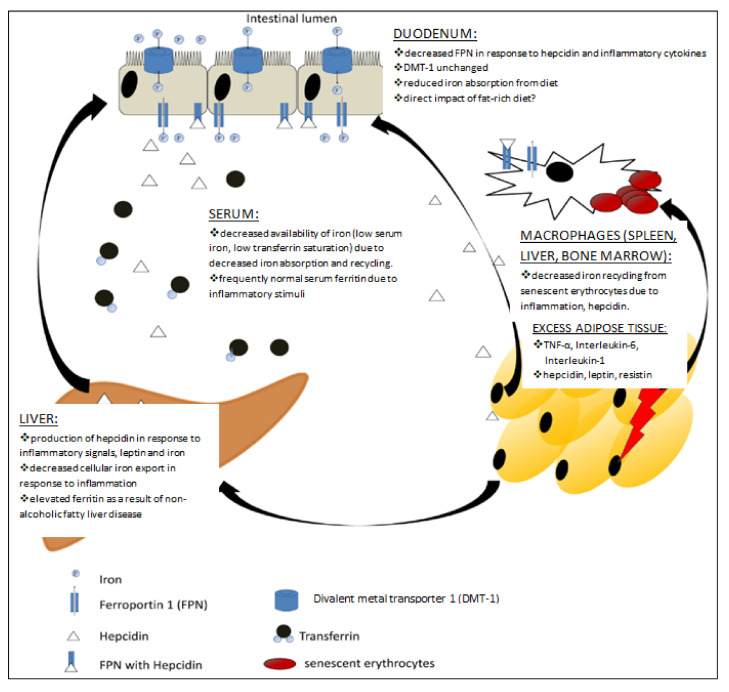
Interplay between obesity and iron metabolism. Figure adapted from Aigner et al. [82].

Several studies have investigated the association between obesity and ID/IDA [57,77,78,79,80], (Figure 2). More obese children are anaemic compared with their non-obese counterparts (58.3% vs. 6.7%) [81] when IDA is defined as a haemoglobin level more than 2 standard deviations below the mean score for age and gender. As described by Nead et al. [78], children who were overweight or at risk of being overweight (BMI of ≥95th and BMI of ≥85th of age using gender-specific percentile, respectively) had double the risk of being iron deficient compared to children with a BMI in the healthy weight range.

### 3.4. Is Inflammation Playing a Role in the Development of ID/IDA in Pregnant Women Who Are Obese?

In an American study of 15 obese and 15 lean pregnant women, levels of hepcidin were higher in the second trimester in obese pregnant women versus non-obese women and were positively correlated with CRP. This observation suggests that the increased inflammatory profile in pregnant women living with obesity is associated with increased hepcidin levels [58]. Moreover, maternal BMI and hepcidin were negatively correlated with cord blood iron parameters (serum iron, TSAT).

A study in pregnant Chinese women [83] (n = 1613) reported 19% as being overweight or obese and 14% as underweight. Women were randomized to receive folate and either placebo or 300 mg of iron sulfate. Maternal blood samples were collected at mid-gestation and at (or near to) term, and cord bloods collected at delivery. Ferritin, sTfR and zinc protoporphyrin/haem were assayed together with CRP as a marker of inflammation. Results indicated that maternal pre-pregnancy BMI was positively associated with CRP in mid- and late pregnancy. In mid-pregnancy, reduced iron status (lower sTfR and body iron (BI)) was associated with higher BMI whereas in late pregnancy there was no association. The authors measured only one marker of inflammation—CRP. Therefore, they were unable to identify participants with all stages of inflammatory state (incubation, early and late convalescence) and could not correct ferritin values for inflammation [84,85]. These ferritin values were subsequently used to calculate body iron. The observed less severe decline in body iron throughout the pregnancy in obese vs. non-obese women could have resulted from ferritin elevation due to inflammation and would therefore not reflect true iron stores. These results may suggest an increased inflammatory response (as evidenced by the increased CRP levels) in obese women and potentially increased hepcidin levels. This in turn works to reduce iron availability for both the mother and her baby. It is also important to note that women who were overweight/obese at the study entry (mid-pregnancy) gained more weight during gestation than lean women. Excessive weight gain during pregnancy was negatively associated with infant iron status.

In a study of 245 pregnant women living with obesity and 245 normal-weight pregnant women, Flynn et al. [86] reported significantly elevated CRP and IL-6 levels in obese vs. lean pregnant women, with no difference in serum ferritin or serum hepcidin levels at 15–18 weeks gestation, suggesting that at least at this stage of pregnancy maternal hepcidin is not driven by inflammation. It is worth noting that obese women had significantly higher sTfR levels and the ratio of sTfR and ferritin when compared to lean women at 15–18 weeks gestation. The authors do not mention if they corrected ferritin values for inflammation [84,85]. Ferritin is an acute-phase protein and elevated CRP and IL-6 in obese mothers is indicative of inflammatory state. Therefore, ferritin values could have been elevated due to the inflammatory state. This might explain the lack of difference in ferritin between obese and lean women. Additionally, since the data were derived from two different studies they differed in maternal characteristics.

Cao et al. [87] assessed the association between pre-pregnancy BMI, gestational weight gain and maternal and neonatal iron parameters (serum ferritin, sTfR, iron, EPO, hepcidin and body iron content). In total, 230 pregnant adolescent women were included from two different cohort studies, with 38% of women falling into the overweight or obese categories. Participants in this study were considerably younger than in other reports, with an age range between 13 and 18 years. During pregnancy, the majority of women (93%) gained more than the recommended weight. The authors collected maternal blood samples at mid-gestation and at delivery together with cord bloods. At mid-gestation pre-pregnancy, BMI was positively associated with inflammatory markers (CRP and IL-6) and with leptin, in line with previous findings [88]. At delivery, however, only the association between pre-pregnancy BMI and leptin remained. There were no significant differences reported between the pre-pregnancy BMI categories and iron status parameters (serum ferritin, sTfR, serum iron, EPO and hepcidin) at mid-gestation or at delivery. However, hepcidin and serum ferritin were significantly higher at mid-gestation in women with class 2 and 3 obesity (BMI 35–39.9 and ≥40 kg/m^2^, respectively) when compared with lean women. Further, pre-pregnancy BMI was positively correlated with serum hepcidin at mid-gestation. Overall, the authors suggest that, in their study, inflammation is not influencing iron metabolism in pregnancy and that perhaps a certain threshold of obesity induced IL-6 has to be reached to affect hepcidin levels.

Garcia-Valdes et at. [89] conducted a study in pregnant women without gestational diabetes (n = 240; n = 90 with normal BMI, n = 37 overweight and n = 31 obese) collecting blood samples at 24, 34 weeks and at delivery. The authors reported that obese mothers had lower iron stores (serum ferritin) at delivery and higher hepcidin levels throughout pregnancy when compared to women with normal BMI. Maternal ferritin correlated with sTfR across all BMI groups and did not correlate with CRP suggesting that, in this cohort, ferritin levels were associated with iron status rather than with inflammation. Interestingly, from approximately 25 weeks gestation onwards, ferritin levels in obese women fell and remained low until term, whereas ferritin levels in normal-weight women increased from approximately 35 weeks gestation till term. The authors suggest that since hepcidin was significantly correlated with CRP, the inflammatory pathway likely plays a part in controlling iron status in obese pregnant women. However, other mechanisms such as production of hepcidin by adipose tissue may also be playing a role.

### 3.5. Can Physical Activity Counteract the Negative Effects of Inflammation in Obese Individuals?

Many pregnant women do not meet the guideline recommendations for level of physical activity [90]. Pregnant women who wore an accelerometer for seven days (n = 359) mostly engaged in inactive tasks and did not meet the recommended levels for physical activity. The lowest activity levels were recorded in the third trimester. This has important implications, as exercise in pregnancy has been associated with lower levels of CRP [91].

Hawkins et al. [92] investigated the association between physical activity and CRP across pregnancy (n = 294) and found that light-intensity physical activity was negatively associated with CRP in the second trimester only when adjusted for BMI, age, smoking status and history of adverse pregnancy outcomes. Tinius et al. [93] reported that in their study of obese third-trimester pregnant women (n = 32), those who engaged in low-intensity but not moderate-intensity activities, had significantly lower levels of CRP when compared to obese inactive women. Both above studies suggest that light-intensity activity can reduce inflammation in pregnant women living with obesity.

Renault et al. [94] investigated whether dietary intervention combined with increased activity would have a dampening effect on inflammation. Pregnant women living with obesity (n = 425, BMI ≥ 30 kg/m^2^) were asked to adjust their lifestyle by implementing the goal of 11,000 steps per day and through the provision of dietary advice (low-calorie Mediterranean-style diet). Women were split into three groups: control, physically active and physically active with dietary advice. Food frequency questionnaires (360 items) were used early and late in pregnancy (at 11–14 weeks and 36–37 weeks, respectively) to evaluate the diet. Women in both intervention groups had lower levels of high-sensitivity CRP (hsCRP) than in the control arm, with the lowest levels seen in women in the physical activity + dietary intervention arm. The authors found that pregnant women who adhered to the exercise goals had hsCRP levels 21% lower when compared to women who did not adhere to the routine. Interestingly, there was a positive association reported between dietary intake of carbohydrates and glycaemic load at 36–37 weeks gestation with hsCRP (measured in weeks 28–30), with women who consumed a high carbohydrate diet having 29% higher hsCRP when compared to women recording the lowest intakes. This study further adds to the body of evidence that light-intensity activity can reduce the inflammatory profile in pregnant women living with obesity, especially when combined with dietary intervention.

Finally, a study reported by Wang et al. [95] suggests that pre-pregnancy exercise may be sufficient to reduce inflammation in pregnancy. A total of 537 women (BMI mean of 24.5 (4.3), 17th week of gestation) self-reported their pre-pregnancy and second trimester physical activity levels. The authors found that vigorous or recreational exercise before pregnancy, but not during pregnancy, was associated with a reduced inflammatory profile during pregnancy.

### 3.6. Risks and Complications Associated with Anaemia in Pregnancy May Be Compounded by an Increased BMI in the Overweight/Obese Range

Anaemic women are at increased risk of antepartum and postpartum haemhorrage [96,97], among other pregnancy complications. Overweight/obesity is also a risk factor for these morbidities, as well as pre-eclampsia, uterine atony and macrosomia [98,99,100,101,102,103]. Pregnant women who are living with overweight or obesity are also more likely to undergo instrumental or caesarean section delivery where the average expected blood loss is approximately double that of an normal vaginal delivery [104,105]. This blood loss increases the risk of anaemia.

**Key findings:**

• Iron deficiency and iron-deficiency anaemia in pregnancy remain a widespread issue [2].
• Obesity is prevalent in the obstetric population and is projected to increase even further [30,31,32].
• A pro-inflammatory profile is observed in obese pregnancy (Table 3) [76].
• Women who are overweight/obese in the first trimester tend to gain more weight during gestation than lean women [83].
• Pre-pregnancy obesity is an independent risk factor for postpartum anaemia [54,55].
• Obese mothers had lower serum ferritin and higher hepcidin levels when compared to women with normal BMI. Maternal ferritin did not correlate with CRP across all BMI groups suggesting that, in this cohort, ferritin levels were likely reflecting iron status rather than inflammation.
• In young mothers (13–18 years of age) with class I obesity, inflammation does not appear to exert control over iron metabolism in pregnancy [87].
• Pregnant women are not meeting the guidelines for physical activity [90].
• Low-intensity exercise has the potential to significantly reduce CRP levels in pregnant obese women with a low-calorie Mediterranean-style diet, lowering the CRP levels further [92,93,94].
• There is potential to use low-carbohydrate diets to reduce the levels of CRP in obese pregnancy [94].
• Overweight/obese mothers are at greater risk of developing ID/IDA due to the metabolic effects of inflammation, complications and higher rate of obstetric interventions that may lead to greater blood loss [102,104,105].

## 4. Effect of Maternal Overweight/Obesity on Infants’ Iron Status

Excessive adipose tissue not only has negative consequences for the mother, but it also may negatively affect the health of the baby. Proteomic studies of placentas from obese and lean mothers have revealed differences in eight proteins that are involved in cellular functions including inflammation, oxidative stress, cytoskeletal structure, growth regulation and others [106]. The authors noted that ferritin was under-expressed in placentas from obese mothers, which may potentially lead to increased levels of free iron, and increased formation of reactive oxygen species and oxidative stress in women living with obesity. Furthermore, due to insufficient placental ferritin expression, iron transfer from mother to fetus may be reduced. Further research is needed to examine this more closely.

Garcia-Valdes et al. [89] reported that, irrespective of maternal BMI, placentas of iron deficient mothers had higher expression of placental transferrin receptor (pTfR 1), which is critical for iron transfer to the fetus. This investigation included 86 placentas and 97 cord bloods from 61 women with normal weight, 20 women in overweight BMI category and 16 women living with obesity. This study also found maternal hepcidin at delivery was strongly correlated with cord blood hepcidin but neither maternal nor cord blood hepcidin correlated with any of the other iron parameters (serum iron, ferritin, TSAT, transferrin, sTfR) of the newborn. Further, there was no difference between cord blood hepcidin levels across the pre-pregnancy maternal BMI groups. Maternal iron status had no effect on ferritin, sTfR and the ratio of sTfR to ferritin in cord bloods. This suggests that the placental increase in pTfR 1 with iron deficiency may play a key role in ensuring sufficient fetal iron levels.

Maternal obesity increases the risk of fetal macrosomia (birth weight above 4500 g) [39]. This increase in birth weight appears to be mainly due to the increase in fat mass [107]. Higher fat mass at birth may contribute to obesity later in life [40,107]. This in turn may lead to low-grade inflammation, a rise in hepcidin levels and, over time, it may result in anaemia of inflammation.

There are reports in the literature concluding that cord blood ferritin levels below 76 µg/L are associated with significantly lower fine motor, tractability, and language skills [108,109]. Similar levels have been reported from China [109], where a ferritin value of 75 µg/L is regarded as low. Two further studies agree that cord blood ferritin concentrations below 76 µg/L are suboptimal and have been associated with negative neurodevelopmental outcomes [110,111].

Flynn et al. [86] reported that babies born to obese mothers had markedly lower cord blood ferritin levels ( <76 µg/L) vs. children of lean mothers. This relationship, however, was no longer evident when adjusting for gestational age at delivery, parity, mode of delivery, maternal smoking status and ethnicity. Interestingly, ethnical differences were noted, with babies born to Black mothers having the lowest cord blood ferritin values when compared to other ethnic groups and White mothers (86.1, 96.1 and 155 µg/L, respectively).

Similarly, a study [112] of 85 pregnant women (n = 34 BMI < 30 and n = 51 women BMI ≥30) found that higher maternal BMI at the time of birth was negatively associated with cord plasma ferritin and positively associated with CRP levels. Umbilical cord hepcidin was unrelated to maternal BMI or maternal inflammatory status. It was, however, negatively associated with haemoglobin and positively associated with ferritin levels. When authors applied a BMI cut off ≥35 (n = 16), inflammatory markers (CRP, IL-6 and TNF-α) in cord bloods were significantly higher than for women with BMI < 35, with diabetes further exacerbating the inflammatory response. These findings support the growing body of evidence that obesity in pregnancy can trigger an inflammatory response and in turn negatively affect iron status of the newborn. 

Conversely, Cao et al. [87] reported that babies born to adolescent obese mothers had significantly higher cord blood body iron levels and haemoglobin when compared to lean mothers. None of the other iron status indicators measured was significantly associated with gestational weight gain or pre-pregnancy BMI. In this cohort, however, almost one-quarter of all infants were anaemic, with a further one-quarter being iron deficient (serum ferritin < 76 µg/L). The high incidence of infant anaemia and ID in this study may be indicative of other factors, such as inadequate dietary iron intakes, that negatively affected the iron status of the neonates.

Korlesky et al. [36] analysed ferritin, hepcidin EPO, reticulocyte(RE)-ZnPP/H (erythrocyte zinc protoporphyrin/haem) and CRP in 201 cord bloods of newborns at greater risk of ID/IDA (such as maternal IDA, pre-gestational diabetes, gestational diabetes, small or large for gestational age infants, low socioeconomic status, ethnic minority) [113]. Approximately 40% of mothers with BMI ≥ 30 and 37% of mothers with BMI < 30 had IDA at the beginning of pregnancy. A further 36% and 23% of obese and non-obese mothers, respectively had gestational diabetes. The authors reported that in newborns from mothers living with obesity ferritin and hepcidin were lower, whereas EPO, haemoglobin and ZnPP/H were higher when compared to newborns of lean mothers. Maternal BMI at delivery was positively associated with newborns EPO and negatively associated with hepcidin, indicating an iron deficient profile in the neonates. 

MacQueen et al. [114] screened 180 cord bloods of high risk-infants (small for gestational age, very low birth weight infants and infants of diabetic mothers) for iron deficiency (ferritin <50 mg/dL, serum iron <100 µg/L and transferrin saturation <30%) and found that all 16 newborns who had ID were born to mothers living with obesity, equating to 4.9-fold higher risk of developing ID compared to if BMI was less than 30. It is worth noting that none of the mothers in this study were anaemic but four had low iron status. The authors suggest that iron transport at the placenta interface might be negatively impacted in overweight/obese pregnancies, a hypothesis that should be investigated further. Similarly, Phillips et al. [115] reported that maternal obesity and excessive weight gain during pregnancy reduced neonatal iron status. The authors measured serum ferritin, transferrin and iron as well as ZnPP/H in 316 newborns at risk of IDA (having at least one risk factor [113]). Pre-pregnancy obesity was present in 28.5% of women, with a further 27.5% exceeding the recommended weight gain in pregnancy. Cord serum ferritin levels were significantly lower while haemoglobin was significantly higher in babies born to obese mothers. Cord serum CRP was not associated with maternal obesity. CRP was, however, associated with an excessive weight gain of 18 kg. Based on their study, the authors conclude that pre-pregnancy obesity and excessive gestational weight gain could be regarded as a risk factor for ID profiles in newborns.

Similarly, in the Chinese study [83] infants born to overweight mothers had a higher incidence of ID (serum ferritin below 75 µg/L, measured in cord blood) when compared to infants born to mothers with normal weight (33% vs. 26%). Neonatal iron status (serum ferritin, body iron) was negatively correlated with maternal pre-pregnancy BMI. Further, sTfR levels were significantly higher and ferritin levels significantly lower in cord blood of neonates born to women with higher BMI.

**Key findings:**

• Lower ferritin expression in placentas from obese mothers may lead to increased levels of free iron, increased formation of reactive oxygen species and oxidative stress. Additionally, insufficient placental ferritin expression may lead to reduced iron transfer from mother to fetus [106].
• Higher maternal BMI at the time of birth is negatively associated with cord plasma ferritin [86,112], and positively associated with CRP levels [112], (Table 4).
• Babies born to young obese mothers (BMI ≥ 30 kg/m^2^) had significantly higher body iron levels and haemoglobin when compared to lean (BMI range between 18.5 and 24.9 kg/m^2^) mothers [87]. Conversely others reported that maternal obesity and excessive weight gain during pregnancy negatively affect neonatal iron status [36,58,83,114,115] indicating an iron deficient profile.
• Placenta’s adaptation (increased level of pTfR 1) may play a key role in ensuring sufficient levels of fetal iron across different groups of pre-pregnancy maternal BMI [89].

## 5. Iron Status in Pregnancy and the Effect of Dietary Interventions and Iron Supplements

A normal adaptation in iron metabolism is observed with pregnancy. For example, during healthy, uncomplicated pregnancies, hepcidin levels are lower compared to non-pregnant women [116]. Barrett et al. [117] have also reported an increase in iron absorption from foods during normal pregnancy, especially during the second and third trimester. Similarly, hepcidin levels are lower in the third than in first or second trimesters [49], in line with an increased need for iron in later pregnancy [5]. This is likely to ensure that increased iron requirements are met during pregnancy [5]. These findings suggest that a diet rich in bioavailable iron should meet increased iron requirements during pregnancy assuming that sufficient iron stores (300–500 mg) are present at the start of pregnancy [6]. However, more iron may be needed if maternal iron metabolism becomes affected by inflammation or genetic disorders. Maternal obesity has also been proposed as another risk factor for poor iron metabolism. A review of iron deficiency in women living with obesity reported impaired absorption of dietary iron associated with elevated hepcidin levels and low expression of duodenal ferroportin as the leading cause of ID/IDA [82]. 

In a study characterising dietary intake of pregnant women in Mexico [88] (normal weight n = 53, obese n = 40; iron deficient n = 9 at study entry), total daily iron intake was calculated based on 24 h diet recalls of four study days. Women in this study were provided with 30 mg of elemental iron supplementation and adherence monitored. Total iron intake was then used as a covariate in the generalized linear model to investigate differences in iron status between the two study groups. A range of iron status markers was measured, including hepcidin, sTfR, ferritin, serum iron and haemoglobin, together with inflammatory markers (CRP, IL-6 and leptin). Pre-pregnancy BMI was not associated with any of the iron status measures. However, it was positively correlated with all of the inflammatory markers. At the first prenatal study visit (13 weeks gestation) ferritin was positively associated with leptin and hepcidin. At the final study visit (approximately 34 weeks gestation), in normal-weight women, all iron status markers were associated with hepcidin, but not with inflammatory markers. There were, however, significant differences between hepcidin (higher in obese compared to normal-weight women), serum iron and all the inflammatory markers (lower iron and higher inflammatory markers in obese compared to normal-weight women). This pattern of associations where higher BMI is associated with higher hepcidin and lower free iron in the presence of inflammation mimics that observed in obese non-pregnant individuals, where adipose tissue appears responsible for the hepcidin upregulation. The authors conclude that low-grade inflammation is likely one of the many components that influence hepcidin levels in pregnant women with obesity. An earlier study from this group supports these findings [118]. 

Koenig et al. [119] used a stable isotope technique to demonstrate that pre-pregnancy obesity does not affect iron utilisation in the third trimester. Data from 50 women (21 pre-pregnancy obese and 29 non-obese) were included in the analysis, and no significant differences were reported in hepcidin levels, corrected serum ferritin, corrected sTfR, haemoglobin, TSAT, EPO, IL-6 or anaemia prevalence when women were split by pre-pregnancy BMI. However, women with a pre-pregnancy BMI in the obese category had significantly higher hsCRP and body iron (corrected for inflammation) when compared to lean women. Pre-pregnancy BMI was positively correlated with IL-6 and hsCRP. More importantly, there was no difference reported between obese and normal-weight women in the third trimester when dietary iron absorption was investigated using ^57^Fe-labelled ferrous sulfate solution. 

The question of whether adequate nutrition during the first 9 months after birth can counterbalance iron deficiency during gestation is important. A review by Radlowski and Johnson suggests that in spite of sufficient iron in the diet, infants born to ID mothers are still ID at 9 months, suggesting that gestational ID negatively affects iron metabolism of the baby beyond 6 months, and potentially longer [21,120,121].

**Key findings:**

• Impaired absorption of dietary iron may be caused by elevated hepcidin [82] and contribute to ID/IDA in overweight/obese pregnant women.
• In women who received iron supplementation throughout pregnancy:
  ○ Hepcidin, CRP and leptin were significantly higher in obese pregnant women compared to lean women, and serum iron was significantly lower;
  ○ Inflammatory markers (CRP, IL-6 and leptin) were not associated with hepcidin;
  ○ Pre-pregnancy BMI was not associated with iron status [88].
• Infants born to ID mothers are more likely to remain ID beyond 9 months of age despite an iron rich diet [21].

## 6. Diagnostics—Correcting Iron Status for Inflammation

It is well established that inflammation or infection confounds iron status indicators (ferritin, hepcidin and to certain degree also sTfR) [69,122,123,124,125,126]. Hence, it is of crucial importance to determine whether inflammation is present when interpreting iron status. This is particularly important in populations where inflammation is common, including pregnant women who are living with overweight or obesity.

Three different strategies have been suggested in order to estimate the iron status of populations with widespread presence of inflammation [85,127]. These are:

• Demonstrating the presence of inflammation with the use of inflammation biomarkers (CRP and AGP) and:
  ○ For research purposes, exclusion of the data, or,
  ○ For clinical practice, careful interpretation of the data obtained from participants with elevated inflammation biomarkers.
• An increased ferritin cutoff for identifying ID from the commonly used 12–15 µg/L [128] to 30 or even 70 µg/L in the presence of human immunodeficiency virus (HIV) [129].
• Utilisation of inflammation biomarkers (CRP and AGP) to adjust the data to allow for the degree of inflammation [84,85,130].

Ferritin is an acute-phase protein which increases with inflammation. Increasing ferritin cutoffs to correctly identify individuals with ID is a possible solution. However, application of an increased cutoff for one population may not necessarily be appropriate for another, due to potential population differences and variances in analytical techniques [131]. Therefore, the use of inflammation biomarkers to adjust ferritin values is potentially the most appropriate approach. 

Namaste et al. [85,130] have suggested using a regression correction to adjust ferritin concentrations using CRP and AGP and malaria infection as the explanatory variables. Similar adjustments may be possible in different populations addressing a range of inflammatory conditions; however, this will require research with large study samples and validation in different populations. 

**Key findings:**

• There is a clear need to employ an appropriate diagnostic method to correctly identify the presence of ID/IDA, particularly in presence of inflammation and to match iron repletion interventions accordingly. This may be possible through future routine analysis of inflammatory markers and ferritin.
• Future research is required to develop algorithms that can be used as diagnostic tools, particularly where inflammation is present.

## 7. Clinical Guidelines and Strategies to Diagnose and Correct ID/IDA in Pregnancy

Standard etiquettes and adherence to protocols have been highlighted as issues in routinely and accurately diagnosing ID/IDA in antenatal and postnatal clinics. For example, large variations in routine iron status assessments have been demonstrated in one region of New Zealand covered by 21 midwifes who provided care to 189 women (with haemoglobin below 110 g/L and/or ferritin below 20 µg/L any time during pregnancy/postpartum period) [132]. Women who were living with overweight or obesity represented 44% of the sample. Out of the whole study cohort, the dietary advice was only provided to 10% of women in the first trimester. Haemoglobin was the only iron status variable consistently measured at the first antenatal visit, with ferritin only measured in approximately half of the women and CRP measured sporadically. This is alarming as approximately 74% of women were iron deficient or anaemic in the second trimester. Out of women who had low iron status identified at any point during pregnancy, only 64% were prescribed/recommended iron supplementation. Unaddressed ID resulted in approximately 47% of women approaching birth with no iron reserves and just under 20% of women entered labour with no known iron status. Significantly lower third-trimester ferritin levels were reported for women with BMI ≥ 25 when compared to women with BMI below 25. The majority of women were not given a haemoglobin test postpartum (77%). This research highlights the need for improvement in standard clinical practice.

Another important issue recently raised by Parker et al. [133] is the lack of uniform guidelines between clinical and laboratory practice for reporting ferritin in North America, highlighting that ferritin reference ranges for women are unacceptably low. This has the effect of masking the real number of women of reproductive age with low iron levels, who then potentially enter pregnancy with insufficient iron reserves. 

A standard clinical approach provided to women living with overweight or obesity may also improve outcomes. Stengel et al. [134] have demonstrated that limited information about gestational weight gain and exercise advice is given to overweight and obese pregnant women. They concluded that antenatal guidelines are very limited, often inadequate and that health professionals unnecessarily discourage women from being physically active.

### Intravenous (IV) Iron as an Alternative to Oral Iron Supplementation in Pregnancy

While a common approach to correct ID/IDA in pregnancy and the postpartum period is oral iron supplementation, this has often proven problematic due to unwanted side effects and limited efficacy, especially during pregnancy [135,136]. Since inflammation impairs gut absorption of iron [137,138], including in pregnancies of women living with obesity [118], the value of using oral iron supplements to correct ID/IDA in obese pregnancy has been questioned. Furthermore, overweight and obese individuals are less responsive to food iron fortification [80], thus this general strategy to improve iron status in the population may not be as successful in pregnant women who are living with overweight or obesity. 

An alternative solution that has increasingly received attention to successfully treat peripartum ID and IDA is the use of intravenous (IV) iron. The development of new IV iron formulations with an improved safety profile made serious adverse reactions a very rare event [139]. This allows for the administration of high doses of iron in a very short period of time [138]. While both oral iron and IV iron have been shown to improve haemoglobin and ferritin profiles during gestation and postnatal period, IV iron provides more rapid and prolonged improvement in the postnatal period [140]. IV iron improved haemoglobin values within 3 weeks of iron infusion, with no adverse drug related impact on the fetus (as measured by fetal heart rate monitoring) [141,142]. It has to be noted, however, that IV iron therapy (especially ferric carboxymaltose infusions) may cause transient hypophosphatemia [143] and, with repeated use, ostemalacia [144].

According to clinical guidelines in the United Kingdom, IV iron in pregnancy is prescribed only when oral supplements are not tolerated, there is non-compliance and/or malabsorption. However, Pavord et al. [145] has recommended that these need to be updated to consider the use of IV iron in ID/IDA pregnant women with oral iron intolerances from the second trimester onwards, especially for ID/IDA women ≥34 weeks gestation. In addition, women with severe anaemia and an increased risk of peripartum haemorrhage should be considered for this treatment option. Australian clinical guidelines are largely in line with the UK guidelines, with stronger emphasis on the use of IV iron if rapid restoration of haemoglobin and iron stores is needed [146]. To date, there have been no studies investigating the effectiveness of different forms of iron treatment modalities, including IV iron formulations, in pregnant women who are living with overweight or obesity specifically. Given the potential lack of efficacy of oral iron formulations with obesity [147,148], tailored IV iron therapy may prove a more beneficial treatment modality for successful and sustained correction of ID/IDA in this population. 

**Key findings:**

• IV iron is effective in improving haemoglobin and ferritin in pregnant women [140,141,142].
• Routine screening processes to identify iron deficiency in the ante- and postpartum periods are needed.
• Procedures for trimester-specific iron supplementation strategies for ID/anaemic pregnant women may help alleviate the current burden of ID/IDA in pregnancy [132].

## 8. Conclusions

There is a growing body of evidence that pre-pregnancy obesity and overweight/obese pregnancies carry greater risk of ID/IDA for the mother during pregnancy and postpartum period [54,55,89] as well as for the baby [21,58,83]. Available studies, mostly conducted in the second trimester, report elevated CRP with elevated or unchanged hepcidin, sTfR and IL-6, suggesting that an inflammatory profile is present in pregnant women with obesity [76] and thus could be playing a role in ID/IDA of overweight/obese pregnancy [89,112]. Further, pre-pregnancy BMI or BMI at the time of birth has been reported to be negatively associated with infant iron status [68,98,101,102]. Mothers who are living with overweight or obesity are at greater risk of developing ID/IDA due to birth complications and a higher rate of obstetric interventions that may lead to greater blood loss [102,104,105].

There have also been studies indicating that inflammation does not have any role in the control of iron metabolism in pregnancy [87] and that babies born to young mothers living with obesity had significantly higher body iron levels and haemoglobin when compared to babies of lean mothers [87]. Adaptation of the placenta (increased level of pTfR 1) could be the key to ensuring sufficient fetal iron levels across different values of pre-pregnancy maternal BMI [89]. However, differences have been reported in placental proteomic data from obese and lean mothers [106], with changes involving cellular functions. 

Finally, no difference in dietary iron absorption has been reported between obese and normal-weight women in the third trimester [119].

The introduction of clearly outlined procedures for trimester-specific iron and inflammatory status assessments during antenatal and postnatal clinics is needed. Tailored treatment strategies depending on the degree of ID/IDA and BMI of pregnant women may reduce the incidence of ID/IDA in pregnancy and the postpartum period. This would potentially reduce the risk of adverse pregnancy outcomes and provide the newborn with an optimal start to life.

Obesity is prevalent in the obstetric population and it is projected to rise even further [30,31,32]. Pregnant women living with obesity also tend to gain more weight during gestation than lean women [83]. Since pregnant women who are obese are more likely to become ID/IDA during pregnancy and the postpartum period [83], a need exists to attend more closely to pre-pregnancy weight and weight gain during pregnancy. Lifestyle changes, such as low-intensity exercise and a low-calorie/low-carbohydrate Mediterranean diet, have the potential to limit weight gain and therefore mitigate the effect of inflammation in pregnancy [92,93,94]. These interventions may therefore have the downstream effect of ensuring an adequate iron status throughout pregnancy.

The overall evidence presented in this review supports the hypothesis that obese pregnant women and their children are more likely to become iron deficient/anaemic both during pregnancy and also in the postpartum period [83].

There are, however, several unanswered questions that can be addressed with future research to optimise the health of pregnant women and their babies.

(i) Do women who are living with overweight or obesity respond differently to iron supplementation?
(ii) Are women who are living with overweight or obesity more likely to enter pregnancy with ID/IDA and thus should they be considered to be at higher risk of developing IDA?
(iii) Does pregnancy dampen the response to inflammation or is there homeostatic adaptation by the placenta and fetus to become more efficient in acquiring iron?

## Figures and Tables

**Table 1:** Estimated iron requirements, absorption and changes in plasma volume and iron status parameters according to pregnancy trimester (table adapted from the Biomarkers of Nutrition for Development review [6] and Bothwell [5]). Changes in levels in the first trimester are relative to the pre-pregnant state.

	Pre-Pregnancy	First Trimester	Second Trimester	Third Trimester	Postpartum, 24 h
Daily iron requirements approx. (mg/d)	1.5	↓ to 0.8 due to halted menstruation	↑ to 4 towards the end of second trim	↑ to 6–10	---
Iron absorption from a highly bioavailable diet ^◊^ (mg/d)	Approx. 1.5	↓ to 0.4	↑ to 1.9	↑ to 5	---
Circulating haemoglobin *	120–160 g/L	↓ by 10 g/L	↓	↑ with Fe supplements ↓ without Fe supplements	↔; individual changes depend on blood loss and fluid shifts.
Anaemia threshold for women Hb (g/L), WHO	120	↓ to 110	110 (WHO) or 105 (CDC ***)	110	---
Red blood cell mass			↑		
Plasma volume expansion, placental growth	---	↑ ** towards the end of the first trimester	↑	↑	---
Serum iron ^a^		↓ **	↓	↓	↔
Serum ferritin ^a^		↓ **	↓	↓	↑ °
sTfR ^a^		↔	↔; ↑	↑, ?	↔

* in the absence of iron deficiency; ** towards the end of the first trimester; ↔ no change; ↓ down; ↑ up; *** CDC—Centers for Disease Control and Prevention, USA. ° increase associated with inflammatory response, correlating with the CRP. ^a^ serum iron, serum ferritin and soluble transferrin receptor (sTfR) are iron status parameters. Hb-haemoglobin; ^◊^ The bioavailability of iron from a highly bioavailable diet is approximately 14% to 18%. These are mixed diets that include substantial amounts of meat, seafood, and vitamin C (ascorbic acid, which enhances the bioavailability of non-heme iron) [7]; ---no data.

**Table 2:** Recommended weight gain ranges in pregnancy according to pre-pregnancy BMI. Source: The US Institute of Medicine [33].

Pre-Pregnancy BMI (kg/m^2^)	Recommended Weight Gain (kg)	Rates of Weight Gain Second and Third Trimester, Average, (kg/wk)
<18.5	12.5–18.0	0.51
15.5–24.9	11.5–16.0	0.42
25.0–29.9	7.0–11.5	0.28
≥30	5.0–9.0	0.22

**Table 3:** Summary of available data with the direction of change of key biomarkers in obese pregnancy vs. normal-weight pregnancy, by trimester.

	Direction of Change in Pregnant Obese Women Compared to Pregnant Normal-Weight Women			
Marker	First Trimester	Second Trimester	Third Trimester	Delivery
Hepcidin [58,87,89]	---	↑↔↑ ↑ *	↑	↔↑
Serum iron [58,87]	---	↔↔	---	↔
TSAT [58]	---	↔	---	---
IL-6 [58,86,87]	---	↔↑↑	---	---
CRP [58,86,87]	---	↑↑↑	---	---
sTfR [86,87,89]	---	↑↔↑	↑	↔↑
ferritin [86,87,89]	---	↔↔↓ ↑ *	↔	↔↓
EPO [87]	---	↔	---	↔
leptin [87]	---	↑	---	↑

* Class II and III obesity; each arrow and its direction correspond to the reported study finding: ↔ no change; ↓ down; ↑ up; ---no data.

**Table 4:** Summarised data specifying direction of change of cord blood parameters in infants born to women living with obesity compared to women with BMI in healthy range.

Marker	Direction of Change of Cord Blood Parameters in Infants Born to Obese vs. Lean Mother
Hepcidin [36,89]	↔↓
Ferritin [36,83,86,112,115]	↓↓↓↓↓
TSAT, serum iron, transferrin [89]	↔
CRP, IL-6 and TNF-α [112]	↑
Body iron [87]	↑
EPO, and ZnPP/H [36]	↑
sTfR [83,89]	↑↔
Hb [36,87,115]	↑↑↑

Each arrow and its direction correspond to the reported study finding: ↔ no change; ↓ down; ↑ up; Hb-haemoglobin

## Abbreviations

ABS	Australian Bureau of Statistics
AGP	α-1 acid glycoprotein
AI	anaemia of inflammation
BMI	body mass index
CRP	C-reactive protein
EPO	erythropoietin
hsCRP	high-sensitivity CRP
ID	iron deficiency
IDA	iron-deficiency anaemia
il-6	interleukin-6
pTfR	placental transferrin receptor
RBC	red blood cells
sTfR	soluble transferrin receptor
TNF-α	tumor necrosis factor
TSAT	transferrin saturation
WHO	World Health Organization
ZnPP/H	erythrocyte zinc protoporphyrin/heme
